# Sharing Neuron Data: Carrots, Sticks, and Digital Records

**DOI:** 10.1371/journal.pbio.1002275

**Published:** 2015-10-08

**Authors:** Giorgio A. Ascoli

**Affiliations:** Krasnow Institute for Advanced Study, George Mason University, Fairfax, Virginia, United States of America

## Abstract

Routine data sharing is greatly benefiting several scientific disciplines, such as molecular biology, particle physics, and astronomy. Neuroscience data, in contrast, are still rarely shared, greatly limiting the potential for secondary discovery and the acceleration of research progress. Although the attitude toward data sharing is non-uniform across neuroscience subdomains, widespread adoption of data sharing practice will require a cultural shift in the community. Digital reconstructions of axonal and dendritic morphology constitute a particularly “sharable” kind of data. The popularity of the public repository NeuroMorpho.Org demonstrates that data sharing can benefit both users and contributors. Increased data availability is also catalyzing the grassroots development and spontaneous integration of complementary resources, research tools, and community initiatives. Even in this rare successful subfield, however, more data are still unshared than shared. Our experience as developers and curators of NeuroMorpho.Org suggests that greater transparency regarding the expectations and consequences of sharing (or not sharing) data, combined with public disclosure of which datasets are shared and which are not, may expedite the transition to community-wide data sharing.

## Abstract 2

Based on his long experience with digital reconstructions of neuronal morphologies, Giorgio Ascoli articulates the benefits and challenges of—and solutions for—changing the cultural resistance to data sharing in the neuroscience community.

Research data are productively shared as a matter of course in many mature domains of science, including molecular biology, particle physics, and astronomy. In neuroscience, in contrast, data sharing is still practiced rarely [1], limiting the opportunity to accelerate the pace of research progress through secondary discovery [2]. Although different subdomains of neuroscience vary somewhat in their attitude toward data sharing [3], widespread adoption of data sharing practice will require a cultural shift in the community [4]. For the past decade, my research team has focused on a particularly “sharable” kind of data, digital reconstructions of axonal and dendritic morphology [5].

The branching structure of axonal and dendritic trees profoundly affects synaptic integration, signal transmission, and network function [6]. Neuronal arbors are imaged with a variety of microscopic techniques in thousands of laboratories worldwide, after staining with a multitude of labeling methods. Neuroscientists trace neurons for multiple reasons, such as establishing neuronal identity; determining potential circuit connectivity; and quantifying morphological changes across development, degeneration, or experimental conditions [7]. Remarkably, neuron reconstructions collected for one purpose can later serve different purposes [8]. Prevalent applications include anatomically realistic biophysical simulations [9], computational models of neural growth [10], and morphometric analyses [11].

NeuroMorpho.Org is a public online repository of digital reconstructions of neuronal morphology [12]. Its primary aim is to enable and encourage open sharing of axonal and dendritic reconstructions from any experimental technique, animal species, brain region, and cell type [13]. We run monthly literature searches to identify all publications that contain new morphological reconstructions and systematically contact the authors to request the data for public posting [14]. When reconstructions are deposited, they undergo a methodical, neuron-by-neuron standardization process [15], which includes (1) conversion to a common non-proprietary file format suitable for visualization, analysis, and modeling; (2) identification and correction of non-controversial tracing errors (such as disconnected subtrees or zero-diameter branches) and annotation (without correction) of other anomalies (e.g., sudden jumps in location); (3) compilation of metadata from original publication and author-provided information; (4) generation of a 2-D static image, a 3-D rotating and growing rendering, and an interactive browser-enabled virtual reality display; and (5) extraction of a comprehensive battery of morphometric parameters [16]. The data are then uploaded to a password-protected review site for quality check. Once the authors are satisfied with the presentation, the neurons are publicly released under the archive name of the corresponding contributors.

The NeuroMorpho.Org web portal allows browsing all entries by archive, species, anatomical region, and neuron type. Furthermore, intuitive query functionalities through drop-down menus or a simple keyword search bar enable filtering and retrieving neurons by a combination of metadata (including the main “browse” dimensions, but also publication reference, histological processing, experimental condition, physical integrity, structural domain, and developmental stage) or morphological characteristics (number of dendritic bifurcations, total axonal length, etc.). Individual neuron pages include all extracted details, built-in visualization, and a PubMed hyperlink to the original article, which in turn links back directly to the available NeuroMorpho.Org data [17]. Downloadable data include the original and the standardized morphological files, the change log, the residual warning log, and all morphometric and metadata information. All morphological and annotation files can also be obtained in bulk from the summary menus.

Since the first release of approximately 1,000 reconstructions nearly a decade ago, the content of NeuroMorpho.Org has increased to over 30,000 neurons, corresponding to approximately 800,000 person-hours of tracing (that is, it would take roughly four centuries for a neuroscientist working full-time to produce an equivalent amount of data). These data are extensively used in the neuroscience community. The site has been accessed more than 200,000 times, and nearly 5 million digital reconstructions were downloaded (Fig 1), amounting to approximately 20 miles of neurite cable. Increasing data availability is also encouraging grassroots development and spontaneous integration of complementary resources [18], research tools [19], and community initiatives [20]. Most importantly, hundreds of peer-reviewed articles were published utilizing data deposited in NeuroMorpho.Org [21]. Dozens of these studies pooled data from many NeuroMorpho.Org archives [22], leading to breakthroughs that would likely have been impossible to obtain by traditionally collecting data in a single laboratory. Even more hearteningly, shared morphological data are now being incorporated in academic education and research training [23].

**Fig 1:**
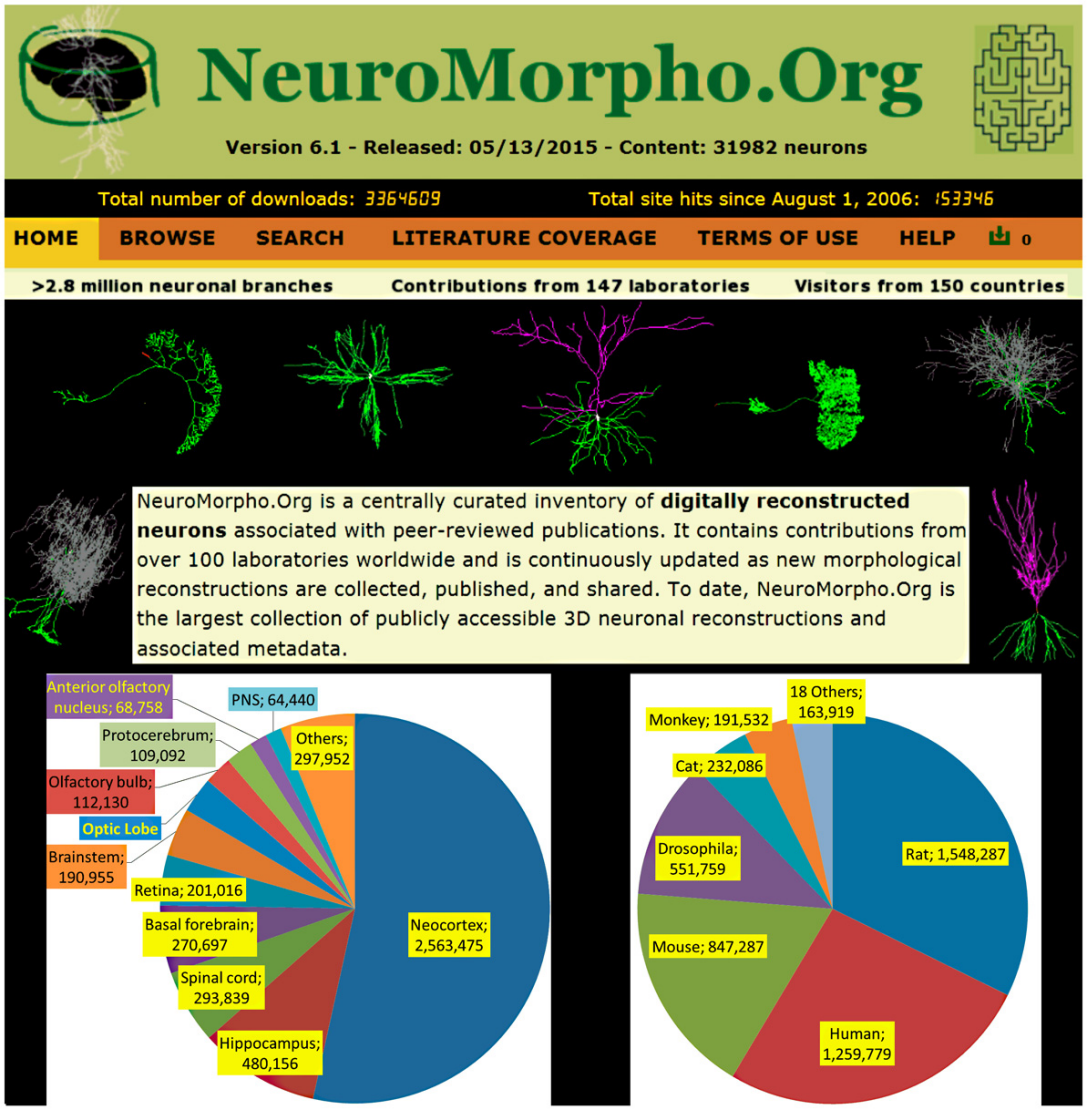
Top: screenshot of NeuroMorpho.Org (captured on July 10, 2015). Bottom: numbers of downloaded reconstructions as of version 6.1 (May 2015) by anatomical region (left) and species (right). In the region pie, “PNS” stands for peripheral nervous system and “Others” include (in order of downloads) mushroom body, basal ganglia, somatic nervous system, visual lobe, adult central complex, ventrolateral neuropils, medulla, antennal lobe, cerebellum, ventral thalamus, amygdala, lateral horn, subesophageal ganglion, hypothalamus, adult subesophageal zone, dorsal thalamus, pharyngeal nervous system, ventral nerve cord, midbrain, stomatogastric ganglion, lateral line organ, pons, and lateral complex. In the species pie, “Others” include (in order of downloads) cricket, salamander, *Caenorhabditis elegans*, elephant, blowfly, goldfish, agouti, sheep, guinea pig, zebrafish, chicken, proechimys, dragonfly, rabbit, turtle, frog, spiny lobster, and moth.

In order to maintain the shared data as a completely open resource, the only requirement for users employing downloaded reconstructions in their work is to cite NeuroMorpho.Org and the original publication(s) that described those morphologies. Since these terms of use rely solely on honor code, we can only track (by citation searches) compliant usage, which provides a lower-bound estimate of impact. For recent examples, we have identified 58 peer-reviewed articles published in the last six months that leveraged NeuroMorpho.Org data. The majority of these publications involve computational modeling, including studies of synaptic integration [24] and plasticity [25], analyses of biophysical [26] and mathematical [27] determinants of activity dynamics, and quantification of morphological constraints on spike propagation [28]. An increasing number of applications pertain to morphometric comparison [29] and classification [30]. In many cases, however, the availability of NeuroMorpho.Org data led to novel, diverse, and unexpected usages, such as estimating irradiation exposure in the nervous system [31], generating unconstrained virtual neuropil surrogates [32], 3-D printing of neuron models [33], and investigating the cellular substrates of diffusion MRI [34].

Although relative to the scant practice of data sharing in neuroscience [35] NeuroMorpho.Org is a resounding success story, fewer than half of the published neuronal reconstructions are shared. The majority of the authors we contact either do not reply or decline the request. What are the roadblocks impeding greater data availability? In the initial phase of this project we experienced a considerable cultural resistance from potential contributors due to their perceived risk of being scooped or otherwise harmed (misinterpreted, proven wrong, etc.) by their own data. Yet after several years and hundreds of examples of successful data sharing through NeuroMorpho.Org, no contributor has ever complained of being scooped, damaged, or feeling sorry for having shared their data.

An increasing reason for refusing to share data in more recent years is lack of time. Although we (database curators) take care of data processing, file formatting, metadata annotation, and quality control, contributors still have to interact with us; discuss and coordinate data sharing with their collaborators, supervisors, or trainees; identify their datasets in the computer labs; and later review the uploaded web posting prior to release. Even though such time investment is negligible compared to the effort already invested to run the original experiments and write the results for publication, it is still a major obstacle to data sharing.

We may, therefore, identify both clear benefits and practical costs of data sharing (Box 1). From the overall research community perspective, the benefits, especially the added potential for scientific discovery [36], far outweigh the costs, mainly time expenditure. However, the comparison is unfairly balanced from an individual’s point of view because in most cases the researchers bearing the cost (data contributors) [37] are not the same researchers reaping the benefits (data users) [38]. In other words, even if a few hours required to share data may save hundreds of hours in new experiments, the scientists who have already spent hundreds of hours in data collection are often unwilling to commit a few additional hours to reduce someone else’s burden. Given the collective benefit of data sharing, clearer incentives must be identified to motivate potential data contributors [39].

**Box 1. Scientific Data Sharing: Benefits and Costs**

**BENEFITS**

The key societal benefit of data sharing is the potential for reuse. Although many positive outcomes of reuse exist, three are particularly noteworthy.

**Discovery**

The most prominent application of shared data is utilization in novel research. It is important to observe that the secondary scientific use of shared data is typically very different from the scope of the primary work: for example, neuronal morphologies originally collected to quantify the effect of different experimental conditions on dendritic branching might be employed, upon sharing, in an anatomically accurate computational simulation of synaptic integration. In fact, some of the possible applications might not be even imaginable at the time of deposition in NeuroMorpho.Org. Furthermore, pooling of shared datasets from multiple studies often enables large-scale data mining analyses that would be unfeasible by traditional single-lab experimental approaches. Moreover, many shared data are employed in numerous independent applications, thus leading to a “multiplicative” (rather than additive) return.

**Reproducibility**

Data sharing allows or facilitates other scholars to reproduce and even expand the original scientific results. While this is a socially delicate issue, the reproducibility of research findings remains of paramount importance to science in general and biomedical investigations in particular. Even though only a minority of the shared datasets undergo attempts to reproduce the original findings, their availability for peer scrutiny instils in and of itself a basic level of confidence against fabrication and trivial mistakes. Moreover, although it is essential for data users and providers alike to remember that no piece of data is ever “perfect,” authors almost invariably double-check their data “one more time” prior to public deposition. This, together with the added value of centralized curation by databases such as NeuroMorpho.Org, yields further quality assurance of shared datasets relative to unshared counterparts.

**Education**

The release of the data underlying a published study, paired with the availability of the publication itself, constitutes a powerful tool for hands-on training. Laboratory courses have long been recognized as essential to academic formation, along with classroom teaching and textbooks. Direct access to data, while not exposing students to the experience of experimental data collection, provides them with the complementary opportunity for first-hand data visualization, manipulation, analysis, and modeling. Linking data to high-performance computational infrastructure through web-based analysis resources will become increasingly important in the world of ever larger data volumes. The continuously growing worldwide impact of massive open online courses (MOOCs) further boosts the educational value of shared digital data in broadly available electronic archives.

**COSTS**

Different kinds of neuroscience data may raise distinct issues that are not encountered in the particular case of neuronal morphology. Nevertheless, the essential cost of data sharing is, in most circumstances, practically ascribable to the necessary investment of the investigator’s time. In our experience, the following are the most commonly perceived culprits:

**Metadata annotation**

To maximize their utility, shared data must be accompanied by a considerable amount of contextual and experimental details that are seldom included in the original peer-reviewed publication, thus requiring investigators to dig into laboratory notebooks. In our perspective, however, such omissions in the published literature should be viewed as troubling in their own merit. From this viewpoint, the process of sharing one’s data ensures completeness of record keeping, a foundation of solid scholarship. A related issue is the time spent in peer-to-peer communication, including initial interactions with database curators and later queries from users. This issue is, in certain ways, similar to the effort necessary to publish a paper at the conclusion of a series of experiments and analyses. Much like a publication potentially reaching thousands of readers, data deposition in a public repository allows researchers to effectively share the results of their work with thousands of potential users.

**Data preservation**

Since NeuroMorpho.Org only stores digital reconstructions of neuronal morphology and not the raw high-resolution image stacks, disk space is hardly an issue. Nonetheless, prior to public deposition of their data, authors often find it necessary to clean up their files, to add comments or explanations, and to create directory summaries. While indeed time-consuming, these same preparatory steps are required to ensure the longevity of one’s data, independent of their public availability. Investigators who choose to avoid this effort all too often find it difficult if not impossible to locate or interpret their own data a decade later. In fact, most researchers who share data in NeuroMorpho.Org end up using this same repository at later times to access their own dataset, taking advantage of the user-friendly web-based retrieval and avoiding the need for maintaining data backups.

It is useful to consider both positive motivations (“carrots”) to reward neuroscientists willing to share their data as well as enforceable rules (“sticks”) to convince peers less inclined towards spontaneous data sharing. The first carrot is the opportunity, recently introduced by several journals and major publishers, to capitalize on the shared data in the form of an additional publication dedicated just to describing the dataset [40].

An additional and particularly appealing built-in incentive is the added impact of shared data. Every time a shared dataset is reused in an independent peer-reviewed scientific report, the primary study of the original data contributor is cited, thus increasing the numerical impact of his or her publication [41]. Moreover, the derivative publication can and should be considered in and by itself objective evidence of the importance of the original study [42]. In these days of increasing hyper-competitiveness, panels evaluating grant proposals, award nominations, academic promotions, and performance raises should no longer rely on mere bean-counting or journal impact factors [43]. Researchers who can demonstrate not only high productivity of their own labs but also the facilitative influence of their data on other labs [44] are well positioned to gain a decisive advantage over similarly productive competitors who choose not to share their data with peers.

To facilitate and stimulate quantitative reporting of shared data usage, NeuroMorpho.Org publishes download statistics at every release and provides data contributors more frequent and detailed updates as needed upon request (e.g., to aid preparation of grant renewals or tenure packets). As of release 6.1 (May 2015), the archive with the largest volume of downloaded reconstruction was Jacobs with 1,114,409 neurons [45], followed by Chiang with 394,933 neurons [46] and by Smith with 335,444 neurons [47]. The archives tallying the most monthly downloads per shared reconstruction are Burke at 51 (each neuron downloaded on average 51 times every month) [48], Cameron at 42 [49], and Fisher at 30 [50]. The number of downloaded reconstructions is only a coarse indicator of the amount of data utilized by the public. Certain neuron types have especially complex arbors, while others have simpler morphologies. Moreover, some reconstructions are more complete or detailed than others. Alternative ways to report data access include weighing every downloaded neuron by its compressed or uncompressed file size, number of tracing points, total length, or number of branches.

Among the most powerful sticks to help maximize the adoption of data sharing are those brandished by funding agencies and peer-reviewed journals. Many grant programs already require inclusion of a data sharing plan in the proposal. However, this aspect of the application is seldom evaluated in depth during the review process. Most importantly, once funding is granted, there are often no mechanisms in place to enforce or even check compliance with the described data sharing plan. At least for multi-year grants, funding agencies could require yearly progress reports to include a description of shared data and an explanation of any deviation from the proposed (and approved) data sharing plan. Similarly, several peer-reviewed journals are now instructing authors to make sharable data available upon publication [51]. However, only for a few data types (gene sequences, protein structures, etc.) are these instructions enforced by requesting a database deposition or accession identifier upon review or prior to accepting the manuscript for publication.

A stricter policing of data sharing compliance by funding agencies and peer-reviewed journals would likely be the most effective force in achieving more comprehensive communal adherence to data sharing practices. An alternative and perhaps simpler path towards the same goal could be to collect and publish data sharing records. The overall societal sentiment is more and more supportive of scientific data sharing [52], and data sharing expectations are being discussed by bioethical commissions in moral and legal terms [53]. When data are invited for deposition in NeuroMorpho.Org, it is increasingly rare for authors to explicitly decline the request [54]. In the majority of cases, authors simply do not respond. This lack of action might feel or appear different from an overt choice not to share, though the effect is identical: those data remain unavailable. Publicly posting the list of not only the shared datasets but also those that have been unsuccessfully requested, makes data unavailability as evident as data availability. Preliminary data suggest that such a step might encourage data sharing.

NeuroMorpho.Org started reporting online the positive or negative outcome of all data deposition invitations since 2012. The proportion of shared datasets increased from one-fifth by 2011 to one-fourth by 2014 and to one-third by the first half of 2015. A substantial number of initially unresponsive authors communicate their willingness to share data just after the third and last reminder, when they are notified that their data would be publicly listed as unavailable. Seminal psychobehavioral studies suggest plausible cognitive explanations for such a phenomenon [55]. In a somewhat similar fashion, the peer-reviewed journal *Neuroinformatics* pioneered in 2004 the instruction to include an explicit statement prior to the Acknowledgments as to whether or not the data pertaining to each article are available [56]. This request ensured more than 90% data sharing rate without a formal policy mandating data sharing. Other journals (including the PLOS family) have more recently adopted similar practices.

Until funding agencies start enforcing stricter data sharing requirements, routine public disclosure of data sharing records could constitute a decisive discriminant between higher- and lower-impact proposals, especially when evaluating competing renewals. These motivators can be particularly useful as temporary catalysts of societal maturation towards spontaneous data sharing. Growing compliance with soft data sharing standards will likely trigger a virtuous chain reaction due to mounting peer-pressure and a natural tendency to follow common practice. In summary, based on our nearly-decennial experience with digital neuron morphology, we recommend and support increased transparency on the benefits of, expectations for, and adherence to data sharing in neuroscience.

The NeuroMorpho.Org experience may not be literally applicable to all domains of neuroscience. Different kinds of data face distinct challenges that, fortunately, do not apply to digital reconstructions of neuronal morphology. Non-invasive whole brain imaging [57] and human genomics [58], for example, raise serious issues of privacy. Electrophysiological recordings depend dramatically on the experimental conditions and vary widely in representation format. For many even less standardized subfields of neuroscience, the lack of a guiding theory and therefore an accepted “code” for the data (compared to, e.g., particle physics, astronomy, and molecular biology) makes it difficult for researchers to converge on common alphabets or languages for data sharing. Nevertheless, we purport that systematically requesting data to be shared while publicly recording the response could catalyze a cultural shift towards data sharing throughout the entire neuroscience community. Following the experience of other established fields of science, we look forward with excitement and optimism to the redoubled discovery impact of neuroscience data by secondary usage and applications.
